# Integrative proteomic profiling of malaria-derived microparticles: A mass spectrometry–based study

**DOI:** 10.5281/zenodo.18327169

**Published:** 2026-01-21

**Authors:** Samuel Antwi-Baffour, Jonathan Kofi Adjei, Francis Agyemang-Yeboah, Max Annani-Akollor, Ransford Kyeremeh, George Awuku Asare, Ben Gyan

**Affiliations:** 1 Department of Medical Laboratory Sciences, School of Biomedical and Allied Health Sciences, College of Health Sciences, University of Ghana, P. O. Box KB 143 Korle-Bu, Accra, Ghana; 2 Department of Molecular Medicine, School of Medical Sciences Kwame Nkrumah University of Science and Technology, Kumasi, Ghana; 3 Noguchi Memorial Institute of Medical Research, University of Ghana, Legon, Ghana

## Abstract

**Background:**

Malaria remains a major public health challenge, causing high mortality and morbidity, particularly in developing countries. Microparticles (MPs), also known as plasma membrane-derived extracellular vesicles (PMEVs), are sub-cellular structures formed by budding off the plasma membrane. Although present in healthy individuals, their numbers increase during pathological conditions such as malaria. While several studies have examined proteins in cell-specific MPs, limited information exists on the protein composition of circulating MPs in malaria and their link to disease symptoms. This study aimed to perform proteomic analyses of MPs from malaria-positive samples, parasite culture supernatants, and healthy controls to elucidate their role in malaria infection.

**Materials and Methods:**

Plasma samples were obtained from forty-three (43) malaria diagnosed patients (cases) and ten (10) healthy individuals (controls). MPs were isolated from malaria parasite culture supernatant and confirmed using flow cytometry. 2D LC-MS was done to obtain their protein content. Resultant data were analysed using SPSS Ver. 21.0 statistical software, Kruskal Wallis test and Spearman’s correlation coefficient r.

**Results:**

In all, 1806 proteins were isolated from the samples. The MPs from malaria-positive samples recorded 1729 proteins, those from culture supernatant 333 while the control samples recorded 234 proteins. The mean number of proteins in MPs of malaria positive samples was significantly higher than that in the control samples. Significantly, higher quantities of haemoglobin subunits were seen in MPs from malaria samples and culture supernatant compared to control samples.

**Conclusions:**

A great number of proteins were observed to be carried in the MPs from malaria samples and culture supernatant compared to controls. The greater loss of haemoglobin from erythrocytes via MPs from malaria patients could serve as the initiation and progression of anaemia in *P. falciparum* infection. Also while some proteins were up-regulated in circulating MPs in malaria, others were down-regulated.

## Introduction

Microparticles (MPs), also called plasma membrane derived extracellular vesicles (PMEVs), are a heterogeneous group of small sub-membrane fragments or membrane-coated vesicles shed from the plasma membrane of various cells during normal cellular activities like growth, senescence, proliferation and apoptosis [1]. MPs carry proteins, lipids and nucleic acids from host cells and are means of intercellular communication and it has been shown that analysis of MPs from blood samples can provide information about the state and progression of a particular disease or condition [1].

Human malaria is caused by five species of the genus *Plasmodium* which is a unicellular protozoan parasite. The disease is a major cause of mortality and morbidity in many developing countries especially in sub-Saharan Africa. An estimated 3.5-4 billion people in 83 countries are at risk of being infected with malaria [2]. Complications associated with malaria infection, particularly in severe malaria, include fever/chills, coagulopathy and anaemia, among other symptoms. At the molecular level, up-regulation of certain cytokines is also thought to relate to malaria-associated high fever [3].

The degree of anaemia experienced in malaria does not always correspond to the parasitaemia level [4]. This is partly caused by a mild bone marrow suppression of erythrocyte production and the collection of complement containing complexes on erythrocyte surfaces after infection which promotes splenic removal of these erythrocytes. Lysis of both infected and uninfected erythrocytes is also considered to be a contributing factor [5]. Severe malaria caused by *Plasmodium falciparum* is associated with the dysregulation of the coagulation system which include endothelial damage, lower levels of anticoagulation and the release of procoagulant MPs [5].

To this end, malaria has been associated with an increase in the level of circulating plasma MPs and plasma concentrations of endothelial MPs (EMPs) which may be proportional to disease severity [6]. The role of infected erythrocyte-derived MPs in cellular communication has been investigated but the protein content (proteomic analysis) of MPs isolated in malaria is yet to be explored [6]. Proteomic analysis on circulating MPs obtained from plasma of malaria-positive blood samples once explored will give a general idea of the protein and protein groups borne by these MPs that may influence the pathophysiology of malaria infection. This study therefore sought to examine the protein composition of plasma MPs of malaria samples and comparing them with proteins of MPs from healthy controls in order to explore their effect on the pathogenesis of malaria and the possible linkage of circulating plasma MPs to malaria anaemia.

Existing literature indicates that elevated MPs levels have been seen in cancer, sepsis, pulmonary hypertension, idiopathic thrombocytopenic purpura and atherosclerosis [5]. Researchers also contend that increased endothelial MP level correlating with disease severity has been seen in malaria [6]. Again, studies in mice models indicate that MPs contributed to induction of systemic inflammation [7]. Others have shown that MPs released after malaria infection, which are primarily erythrocyte-derived, can activate macrophage through toll-like receptors (TLR) and may enhance infectivity as their count elevates and investigations show they contain parasite components some of which promote pathogen invasion of erythrocytes [7].

Regev-Rudzki *et al.* [8] postulated that MPs released in malaria are also capable of activating the blood–brain barrier which exacerbates inflammation [8]. The perplexing feature of malarial anaemia, which is increased clearance of uninfected erythrocytes, can also be attributed to the release of parasite antigens in MPs during entry in erythrocytes. These erythrocyte-adhesive proteins probably adhere to erythrocytes resulting in IgG and complement binding which promotes their elimination from peripheral circulation [9]. Furthermore, Schorey *et al.* [10], stated that MPs released from P. falciparum-infected cells can modulate hosts immune response thereby impairing surveillance.

Proteomic analysis on circulating plasma MPs obtained from plasma of malaria-positive blood samples once explored will give a general idea of the protein and protein groups borne by these MPs thereby influencing the pathophysiology of malaria infection. This study seeks to examine the protein composition of plasma MPs of malaria samples in order to explore their effect on the pathogenesis of malaria and the possible linkage of circulating plasma MPs to the anaemia.

## Methodology

The study was a cross-sectional study conducted over a 2-year period, from May 2014 to June, 2016. The samples were collected at the Sunyani Regional Hospital, Ghana and all the laboratory work was carried out at the Department of Molecular Medicine, Kwame Nkrumah University of Science and Technology (KNUST), Noguchi Memorial Institute for Medical Research (NMIMR) and the Proteomic and Flow cytometry Core Facilities of the Indiana University School of Medicine.

### Study population and sampling

Fifty-three out-patient participants were recruited into the study. There were 43 (23 males and 20 females) malaria-positive cases and 10 (5 males and 5 females) control subjects. The age range for controls was 28–47 years with a mean of 38.1 yrs while the age range for patients was 1 week to 62 years with a mean of 25.0 yrs.

Convenience sampling was employed for this study. Three millilitres (3 ml) of blood was collected from 43 laboratory-diagnosed malaria patients into K_2_EDTA tubes mixed and analysed within 6 hrs. Parasite density was confirmed using independently prepared thick films. Control samples were obtained from 10 apparently healthy individuals. The samples were categorised into mild, moderate and high based on the level of parasitaemia.

### Full blood count for RBC indices

Full blood count (FBC) analysis for haemoglobin and other RBC indices was conducted on the venous blood using a Sysmex automated haematology analyser (Sysmex, Kobe, Japan). The analyser was calibrated according to manufacturer instructions, with daily QC performed. Samples were automatically aspirated and processed. RBCs were measured by electrical impedance and haemoglobin by the SLS photometric method. Haematological indices were derived from primary measurements. Results were checked for instrument flags, with abnormal samples re-run or confirmed by peripheral smear before final data entry.

### Thick films

Briefly, 6 μl of blood was placed in the middle of a glass slide for thick film preparation using the WHO standardised template. The slides were then air-dried and subsequently stained with 1:10 diluted Paskem Giemsa for 10 min, air dried and examined using the Olympus CX 31 microscope (Tokyo, Japan). Parasite count was calculated using the formula:

Parasite count=Number of parasite countedNumber of WBC counted×6000μl

where 6000 is the assumed WBC count per 1 μl of blood.

### Purification of MPs from plasma

Briefly, EDTA-anticoagulated blood (3 ml) was centrifuged at 160 × g for 5 min, and plasma was stored at −20 °C until further processing. During processing, samples were thawed and centrifuged at 4000 × g for 60 min to obtain platelet-free plasma. The resultant supernatant was then spun for 120 min at 19,000 × g to obtain the microparticle pellet after the supernatant had been discarded [11]. Fifty μl of PBS was then added to the MP pellet and the suspension stored at −80 °C.

### Flow cytometry analysis

All reagents used in the flow cytometry experiments were from BD Biosciences unless otherwise stated. The frozen MP suspension was thawed and 50 μl of phosphate buffered saline (PBS) added. Equal volume of Annexin V binding buffer was added to label the MPs. Labelled plasma samples were analysed on a BD FACSAriaTM flow cytometer. MPs isolated from plasma were gated (Annexin V+) based on their forward (FSC) and side (SSC) scatter distribution as compared to the distribution of synthetic 0.7–0.9 μm SPHERO™ Amino Fluorescent Particles (Spherotech Inc. Libertyville, Illinois, US). Taking into account the presence of phophatidylserine (PS) residues in MPs surface, events present in Annexin V+ region were accessed for their positive staining with annexin V (BD Bioscience). Flow cytometry analysis was done to verify the presence of Annexin V+ MPs in the pellet.

### Proteomic analysis

One hundred μl of frozen MPs fractions were thawed and resuspended in 60% methanol and 0.1 M ammonium bicarbonate pH 8.0. Incubation with diothiothreitol followed by cysteine alkylation with iodoacetamide reduced the proteins. The solution was incubated overnight with mass spectrometry grade trypsin (Pierce, USA) at 37 °C to digest the proteins. The obtained tryptic peptides underwent desalting using reversed phase cartridge by washing in 0.5% acetic acid. The peptides were then eluted with 95% acetonitrile and 0.5% acetic acid. The eluted peptides were lyophilised and re-suspended in 0.5% acetic acid and directly analysed using 2-dimensional liquid chromatography tandem mass spectrometry (2D-LC-MS/MS). The samples were loaded onto a micro-capillary strong cation exchange column and fractions were collected using an increasing salt step elution gradient. Afterwards, each fraction was analysed using the LTQ ion trap mass spectrometer (Thermo-Fisher Scientific, Waltham, MA). The acquired MS/MS spectra were searched against protein database available at www.uniprot.org [12]. The protein interactions were acquired using the string database available at http://string-db.org/cgi/.

### Western blotting

MPs were thawed and lysed in sample buffer and were loaded on SDS-PAGE gels. The presence of the identified proteins in the samples was analysed by Western blot. Bands at the predicted molecular weight for the proteins were observed. All experiments (both independent and technical) were repeated 2–4 times.

Membranes were incubated with the following primary antibodies: Anti-GAPDH (Rabbit monoclonal, Cell Signaling Technology, Cat# 2118; dilution 1:1000), Anti-TSG101 (Mouse monoclonal, Abcam, Cat# ab83; dilution 1:500), Anti-Flotillin-1 (Rabbit polyclonal, Cell Signaling Technology or Abcam, Cat# ab41927; dilution 1:1000) and Anti-Hemoglobin A (HbA) (Mouse monoclonal, e.g., Santa Cruz Biotechnology, Cat# sc-21757; dilution 1:500).

The membranes were then incubated with the appropriate HRP-conjugated secondary antibodies: Anti-rabbit IgG–HRP (1:3000 dilution) and Anti-mouse IgG–HRP (1:5000 dilution).

All antibodies were diluted in 5% BSA or 5% non-fat milk prepared in TBST (0.1% Tween-20 in TBS), depending on manufacturer recommendations.

### Data analysis

Proteins identified were analysed under molecular function, biological processes, sub-cellular location and cellular component using the uniprot database. Resultant data were further analysed using SPSS 21.0 statistical software. Differences between the medians of the various groups were analysed by Kruskal Wallis test. Pairwise correlations were evaluated with Spearman’s correlation coefficient r. A p-value < 0.05 was considered to be statistically significant. For illustration purposes the samples were categorised into 3 sub-groups according to the parasitaemia based on the old system of classification (1 +, 2+ and 3+ which corresponded to parasite counts <5000/μl, >5000/μl but <10,000/μl and parasite count > 10,000/μl respectively in this study) to analyse their association with proteins released in MPs.

## Results

Participant characteristics are presented in Table 1. All patients were reporting on an outpatient basis. The age range for controls was 28–47 years while the age range for patients was 1 week to 62 years.

**Table 1:** Participant characteristics.

	Control	Patients
Number	10	43
Female (%)	50	47.5
Male (%)	50	53.5
Median Age (years)	37.5 (28-47)	22 (0.003-62)
Number below 5 years	0	9
Number pregnant	0	4

### Plasmodium species

All patients tested had *P. falciparum* malaria. Subsequently the MPs types seen also carried *P. falciparum* markers such as Merozoite surface protein 1, Actin-1 and Tubulin beta chain as well as those of other activated cells (endothelial cells, erythrocytes, leukocytes and platelets).

### RBC indices

The red blood cell indices showed significant variation between the *P. falciparum* infected patients and the controls. For example, there was significance in haemoglobin concentration, MCV, MCH and RDW (Table 2).

**Table 2:** Red blood cell indices.

Parameter (Unit)	Controls	*P. falciparum* patients	P- value
	Mean + SD	Mean + SD	
Hb(g/dl)	13.20 + 0.94	10.35 + 2.61	0.037
Hct (%)	36.30 + 3.35	30.74 + 8.10	0.184
MCV(fl)	92.95 + 2.37	80.68 +10.75	0.029
MCH(pg)	33.78 + 0.35	26.82 + 3.66	0.000
MCHC (g/dl)	35.75 + 0.64	33.28 + 2.64	0.072
RBC count (xlO12 /l)	3.83 + 0.26	3.86 + 0.83	0.934
RDW (%)	12.05 + 0.29	15.80 + 3.35	0.032

### Proteomics analysis

**Figure 1:**
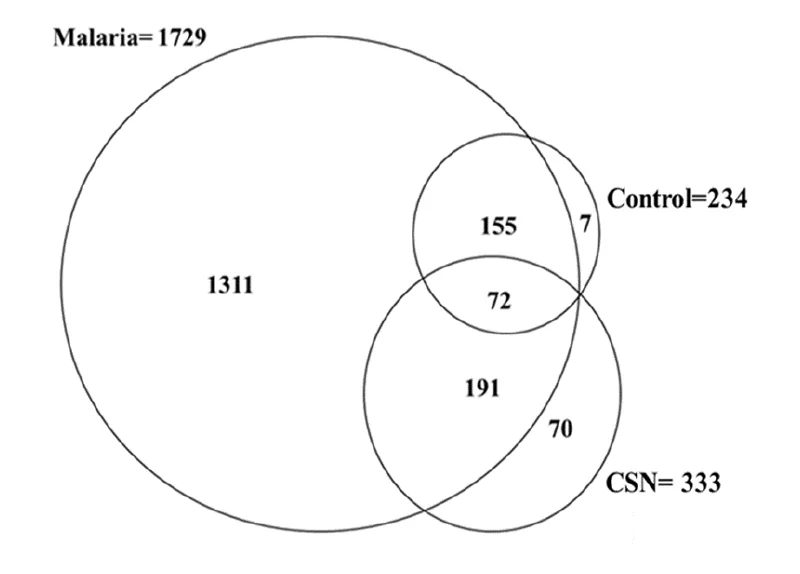
Venn diagram depicting overlapping MP proteins identified in malaria culture supernatant and control samples.

A total of 1729 proteins were identified in the malaria positive samples. The culture supernatant (CSN) showed 333 proteins while a total of 234 proteins were identified in the control samples. Seventy-two (72) proteins were common to the malaria positive samples, CSN and the control (Figure 1).

The mean number of proteins obtained from MPs in malaria samples was 517.58 ± 56.58 while that of control was 191.50 ± 5.20. The independent sample t-test showed significantly higher number of proteins released in MPs from malaria samples. Twenty-nine *P. falciparum* proteins were identified in at least one malaria positive sample (Table 3).

**Table 3:** *P. falciparum* proteins identified in MPs from malaria positive samples.

Accession	Protein Name	Protein MW	Species
P04934	Merozoite surface protein 1	196199.8	PLAFC
P08569	Merozoite surface protein 1	193722.4	PLAFM
PI3819	Merozoite surface protein 1	193721.5	PLAFF
PI9598	Merozoite surface protein 1	192465	PFAF3
P86287	Actin-1	41871	PFAFX
Q8I4X0	Actin-1	41871	PFAF7
PI0988	Actin-1	41843	PFAFO
PI1144	Heat shock 70 kDa protein	74288	PFAFA
Q00080	Elongation factor 1-alpha	49041.2	PFAFK
P06719	Knob-associated histidine-rich protein	71941.5	PFAFN
P14643	Tubulin beta chain	49751.4	PLAFK
Q7KQL5	Tubulin beta chain	49751.4	PFAF7
P38545	GTP-binding nuclear protein Ran	24875.5	PFAFA
Q27727	Enolase	48704.2	PFAFA
Q8IJN7	Enolase	48678.1	PFAF7
Q9UAL5	Enolase	48662.1	PFAFG
P14140	Tubulin beta chain	49814.4	PFAFA
PI3830	Ring-infected erythrocyte surface antigen	124907.8	PFAFF
Q25761	ADP-ribosylation factor 1	20840	PFAFO
Q7KQL3	ADP-ribosylation factor 1	20912	PFAF7
Q94650	ADP-ribosylation factor 1	20912	PFAFA
P12078	Heat shock 70 kDa protein PPF203 (Fragment)	23057.9	PFAFA
PI3816	Glutamic acid-rich protein	80551.3	PFAFF
PI9260	Merozoite surface antigen 2, allelic form 2	28555.5	PFAFG
PI9599	Merozoite surface antigen 2	27890.3	PFAFF
P50490	Apical membrane antigen 1	71968.2	PFAFG
Q03498	V-type proton ATPase catalytic subunit A	68577	PFAFA
P06916	300 kDa antigen AG231 (Fragment)	33968.1	PFAFF
P04928	S-antigen protein	33695.1	PFAFN

Twenty-three (23) RAB proteins were identified in MPs from malaria samples exclusively without any being found in the control samples (Table 4).

**Table 4:** List of Rab proteins identified in MPs isolated from malaria samples.

Accession	Protein Name	Protein MW	Species
Q9H0U4	Ras-related protein Rab-IB	22171.4	HUMAN
000194	Ras-related protein Rab-27B	24608.1	HUMAN
P62820	Ras-related protein Rab-IA	22678	HUMAN
P61006	Ras-related protein Rab-8A	23668.4	HUMAN
P61106	Ras-related protein Rab-14	23897.2	HUMAN
P61026	Ras-related protein Rab-10	22541.1	HUMAN
P31150	Rab GDP dissociation inhibitor alpha	50583.2	HUMAN
P51149	Ras-related protein Rab-7a	23490	HUMAN
Q92930	Ras-related protein Rab-8B	23584.3	HUMAN
Q9NRW1	Ras-related protein Rab-6B	23461.9	HUMAN
P62491	Ras-related protein Rab-llA	24393.7	HUMAN
P50395	Rab GDP dissociation inhibitor beta	50663.7	HUMAN
Q15286	Ras-related protein Rab-35	23025.4	HUMAN
P20340	Ras-related protein Rab-6A	23593	HUMAN
P51148	Ras-related protein Rab-5C	23482.8	HUMAN
P51153	Ras-related protein Rab-13	22774.3	HUMAN
Q13637	Ras-related protein Rab-32	24997.5	HUMAN
Q96AX2	Ras-related protein Rab-37	24815.4	HUMAN
Q96E17	Ras-related protein Rab-3C	25952.4	HUMAN
Q9UL25	Ras-related protein Rab-21	24347.8	HUMAN
P61020	Ras-related protein Rab-5B	23707	HUMAN
P61019	Ras-related protein Rab-2A	23545.8	HUMAN
Q9NP72	Ras-related protein Rab-18	22977.3	HUMAN

**Figure 2:**
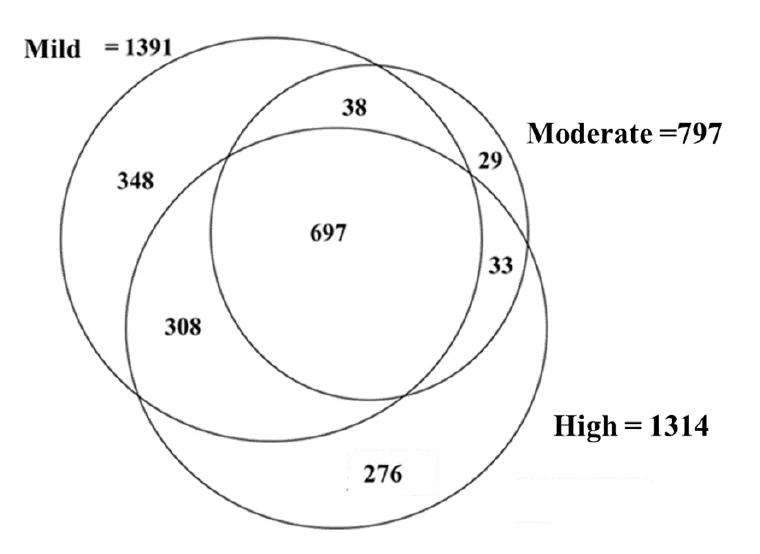
Venn diagram depicting overlap of MP proteins identified in the 3 categories of malaria samples.

Out of the 1729 proteins found in the MPs from malaria positive samples, 653 were identified in plasma MPs from 1 category of samples only while 1076 proteins were identified in at least two categories and 697 proteins were found to be common to all 3 categories. The details of the relationship between the proteins released in plasma MPs from the 3 categories of malaria positive samples are shown in Figure 2.

### Plasma MPs and inflammation

Some of the proteins identified showed strong link to inflammation. Inflammatory proteins seen in malaria MPs but not control sample MPs include Heat shock protein (HSP) 90-alpha, HSP 90-beta, 60 kDa HSP mitochondrial, 70 kDa HSP, HSP beta-1, Transforming growth factor beta-1 induced transcript 1 protein, Macrophage migration inhibitory factor and Transforming growth factor beta-1.

Again, inflammatory proteins released in malaria MPs but in reduced quantities included: Heat shock cognate 71 kDa protein, Heat shock 70 kDa protein 1A/1B, Heat shock 70 kDa protein 6, Heat shock 70 kDa protein 1-like.

### Plasma MPs and the complement system

A good number of complement associated proteins were seen in the MPs from the malaria samples. It was observed that the levels of Complement C3 and Complement C4-B, Complement C5, Complement C9, Complement factor B and Complement C1q subcomponent subunit C were significantly elevated in malaria plasma MPs compared to controls (Table 5).

**Table 5:** Analysis of complement proteins from samples.

Parameter	*P. falciparum* positive	Controls	P- value
N on-Parametric	Median (Q1-Q3)	Median (Q1-Q3)	
Complement C3	192.5 (181-204)	134 (88-183)	0.083
Complement C4-B	103 (96-110)	38 (23-60)	0.000
C4b-binding protein alpha chain	18.5 (18-19)	15 (10.25-19.75)	0.192
Complement Clr subcomponent	15.5 (12-19)	16.5 (6.75-24.25)	0.895
Complement Cls subcomponent	9(5-13)	6 (4-11.75)	0.311
Complement C5	11(9-13)	64(2-8)	0.620
Complement Clq subcomponent subunit B	3.5 (3-4)	5 (3-7)	0.142
Complement component C9	23 (21-25)	11 (7-18)	0.000
Complement factor B	11.5 (8-15)	5 (3-9.75)	0.025
Complement factor H	12.5 (5-20)	5 (2-10)	0.150
Complement Clq subcomponent subunit A	2 (1-4)	3(2-5)	0.315
Complement Clq subcomponent subunit C	4(2-6)	2 (1-3)	0.035
Complement component C7	6(4-8)	2 (1.75-4)	0.061
Parametric	Mean + SD	Mean + SD	
Complement component C6	4.27 + 0.43	1.33 + 0.52	0.000

### Plasma MPs and haemostasis

The following proteins associated with coagulation were released in malaria plasma MPs but not in control plasma. These are: Thrombospondin-1, Thrombospondin-4, Coagulation factor V, Coagulation factor XII and Coagulation factor XIII A chain. Again, Fibrinogen beta chain and plasminogen released in the malaria plasma MPs were significantly higher compared to control plasmas. There was, however, no significant difference in levels of Fibrinogen alpha chain, Fibrinogen gamma chain and von Willebrand factor between the 2 groups. Antithrombin-III in plasma MPs was, however, significantly reduced in the patients compared to the control group (Table 6).

**Table 6:** Analysis of haemostatic proteins from samples.

Parameter	*P. falciparum* positive	Controls	P- value
N on-Parametric	Median (Q1-Q3)	Median (Q1-Q3)	
von Willebrand factor	15 (5.5-27)	11.5 (11-12)	0.493
Antithrombin-III	5.5 (3-8)	17 (13-21)	0.001
Thrombospondin-1	0	39 (17.25-71.25)	0.000
Coagulation factor V	0	16.50 (5.25-36.50)	0.000
Coagulation factor XIII A chain	0	8 (1-19)	0.000
Thrombospondin-4	0	0.00 (0-3.75)	0.043
Coagulation factor XII	0	1(0-2)	0.004
Plasminogen	0	30.0 (10.25-43.75)	0.000
Parametric	Mean + SD	Mean + SD	
Fibrinogen alpha chain	59.01 + 28.35	88.5 + 0.58	0.045
Fibrinogen beta chain	56.79 + 27.62	74.13 + 6.93	0.225
Fibrinogen gamma chain	37.56 +16.05	53.37 + 9.24	0.066

### Plasma MPs and haemoglobin sub-units

Some haemoglobin subunit proteins were seen in the circulating MPs from both malaria positive samples and controls. The quantities of haemoglobin subunits in plasma MPs from malaria positive samples were, however, significantly higher compared to controls (Table 7).

**Table 7:** Analysis of haemoglobin subunit proteins in samples

Parameter	*P. falciparum* positive Median (Q1-Q3)	Controls Median (Q1-Q3)	P-value
Haemoglobin subunit gamma-2	23 (10-63)	1 (1-1)	0.001
Haemoglobin subunit beta	143 (69-274)	13 (11-15)	0.000
Haemoglobin subunit gamma-1	28 (10.5-64.5)	1 (1-1)	0.000
Haemoglobin subunit alpha	125 (52-284)	11 (9-13)	0.000
Haemoglobin subunit delta	72 (40-138)	3.5 (3-4)	0.000
Haemoglobin subunit epsilon	21(8-51)	1 (1-1)	0.000

### Plasma MPs and adhesive proteins

Some adhesive and receptor proteins were identified in the plasma MPs from the malaria samples but they were absent in the control samples. These included: Merozoite surface protein 1, Knob-associated histidine-rich protein, disintegrin and metalloproteinase domain-containing protein-10,-12,-17, Complement receptor type 1 , Cysteine and glycine-rich protein 1, Ring-infected erythrocyte surface antigen, Glycophorin-binding protein, Intercellular adhesion molecule 2, A disintegrin and metalloproteinase with thrombospondin motif 13, Merozoite surface antigen 2, Endothelial cell-selective adhesion molecule.

### Plasma MPs and cytoskeletal proteins

Cytoskeletal proteins were identified in the malaria samples but these were absent in the control samples. Spectrin alpha chain, erythrocytic 1, Spectrin beta chain erythrocytic 1, Coronin 1A, Coronin 1C, Myosin 9, Actin-1, Actin-related protein 3, Actinrelated protein 2, Actin-related protein 2/3 complex subunit 3, Actin related protein 2/3 complex subunit 4 and Actin-related protein 2/3 complex subunit 5.

### Flow cytometer distribution for MPs show a typical forward-/side-scatter distribution

**Figure 3:**
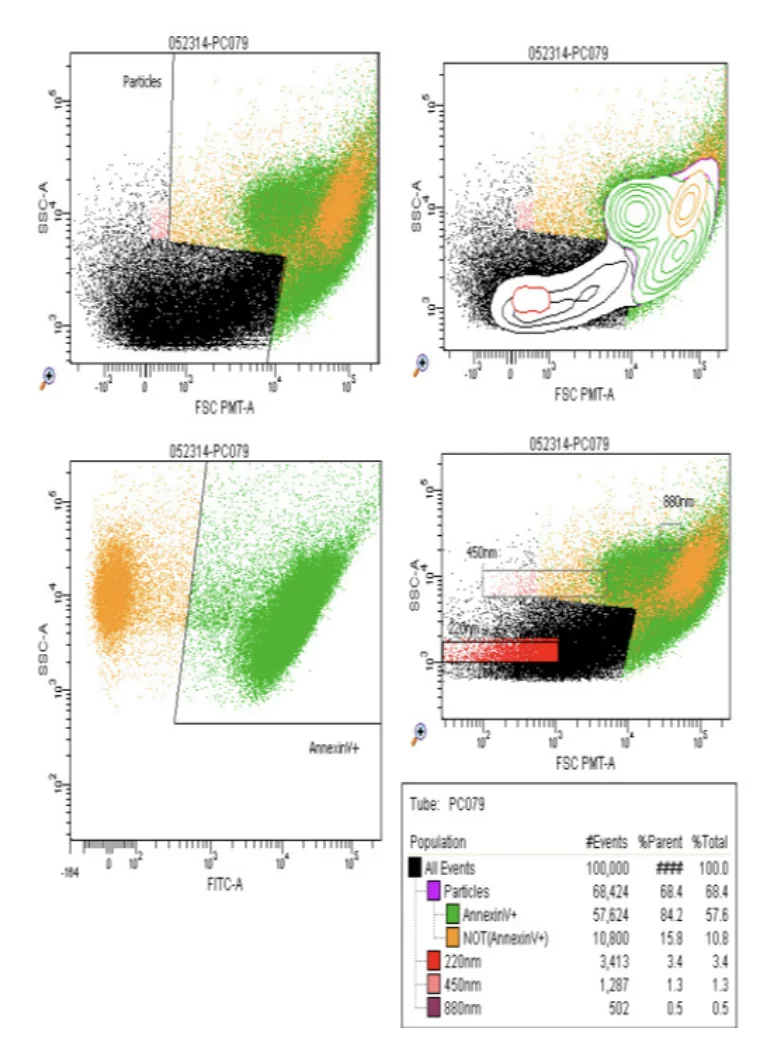
Read out from flow cytometry depicting the classical appearance of MPs in samples.

MPs can be identified by their forward-/side- scatter appearance on flow cytometer. As can be seen from Figure 3, the forward/side scatter distribution of the MPs from the samples shows a similar pattern in line with the classical appearance of MPs on flow cytometer and comparable to those obtained by others in similar experiments. This, in combination with other properties such as the proteins detected give credence to the fact that throughout our experiments, MPs were being obtained.

### Protein enrichment of the isolated MPs

**Figure 4:**
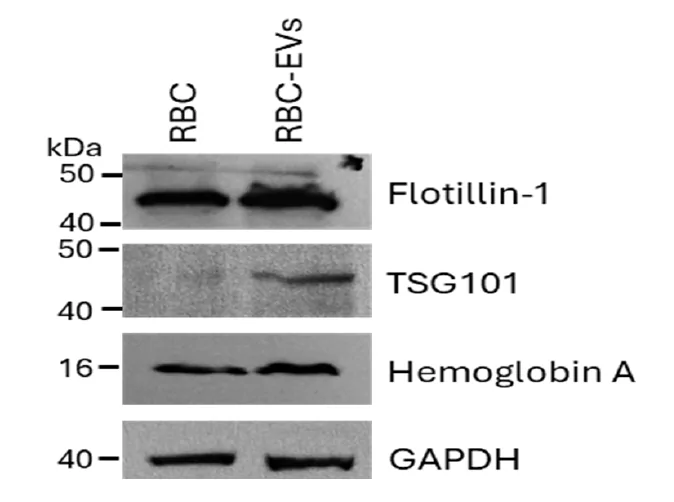
Western blot analysis of MP markers Flotillin-1, and TSG101 and of the RBC marker, haemoglobin A, with GAPDH as a loading control in Red cell lysates and from RBC-derived MPs.

Figure 4 shows protein enrichment of MPs (A) and proteins differentially expressed by MPs (B) by Western blot. The proteins were loaded according to the sample categories of mild, moderate and high malaria as well as no-malaria and bands at the predicted molecular weight for each of the proteins were observed.

### Correlation coefficient of clinical variables

Tables 8, 9 and 10 show the Spearman correlation coefficients of various groups of proteins identified in MPs from malaria samples against the parasite count and peripheral haemoglobin concentration. There was no significant correlation of all the haemoglobin subunits against either the peripheral haemoglobin concentration or parasite count.

**Table 8:** Spearman’s rank correlation coefficients of Haemoglobin subunits and coagulation proteins released in MPs against peripheral haemoglobin concentration and parasite count.

Microparticle Protein	HB	Parasite Count
HB		-0.415**
Haemoglobin subunit epsilon	0.2	-0.25
Haemoglobin subunit gamma-2	0.201	-0.227
Haemoglobin subunit beta	-0.063	0.085
Haemoglobin subunit gamma-1	0.197	-0.17
Haemoglobin subunit alpha	-0.09	0.212
Haemoglobin subunit delta	-0.059	0.13
Haemoglobin subunit mu	-0.5	0.5
Alpha-Haemoglobin-stabilizing protein	0.5	-0.5
Fibrinogen beta chain	0.211	-0.254
Fibrinogen alpha chain	0.029	0.121
Fibrinogen gamma chain	0.285	-0.131
Coagulation factor V	0.177	-0.151
Coagulation factor XIIIA chain	0.053	0.037
von Willebrand factor	0.036	0.047*
Antithrombin-III	0.175	-0.079
Coagulation factor XII	0.2	-0.006

* Significant at P < 0.05; ** same at P < 0.001

**Table 9:** Spearman’s rank correlation coefficients of Complement proteins released in MPs and peripheral haemoglobin concentration and parasite count.

Complement Proteins	HB	Parasite Count
Complement C4-B	0.21	-0.161
Complement component C6	-0.207	-0.414
Complement C3	0.457**	-0.359*
C4b-binding protein alpha chain	0.095	0.103
Complement Clr subcomponent	0.006	0.045
Complement Cls subcomponent	-0.081	0.407**
Complement C5	-0.017	0.025
Complement Clq subcomponent subunit B	0.147	-0.191
Complement component C9	0.045	-0.111
Complement factor B	0.007	0.144
Complement factor H	0.078	-0.004
Complement Clq subcomponent subunit A	-0.071	0.352
Complement Clq subcomponent subunit C	0.124	-0.224
Complement component C8 beta chain	0.202	0.251
Complement component C7	0.025	0.350
Complement factor I	-0.059	-0.736
Complement component C8 alpha chain	0.421	0.089
Complement component C8 gamma chain	-0.344	0.465
Complement decay-accelerating factor	-0.514	0.062
C4b-binding protein beta chain	-0.338	0.845*
Complement factor H-related protein 2	-0.258	0.775
Complement factor I	-0.059	-0.736
Complement component C8 alpha chain	0.421	0.089
Complement component C8 gamma chain	-0.344	0.465

* Significant at P < 0.05; ** same at P < 0.001

**Table 10:** Spearman’s rank correlation coefficients of selected microparticle proteins against Parasite count and Haemoglobin.

Microparticle Protein	HB	Parasite Count
Leukocyte elastase inhibitor	-0.106	-0.206
P-selectinO	0.078	-0.023
Mannan-binding lectin serine protease 2	-0.664**	0.176
Intercellular adhesion molecule 1	-0.686	0.169
Intercellular adhesion molecule 2	-0.093	-0.109
Intercellular adhesion molecule 3	-0.726*	0.676
Mannan-binding lectin serine protease 1	-0.157	0.258

* Significant at P < 0.05; ** same at P < 0.001

Peripheral haemoglobin, however, negatively correlated with parasite count (r = −0.415, p < 0.001) and Mannose-binding lectin serine protease 2 (r = −0.664, p < 0.001) while it positively correlated with Complement C3 (r = 0.457, p < 0.001). Parasite count correlated negatively with Complement C3 (r = −0.359, p < 0.05) and correlated positively with Complement C1s subcomponent (r = 0.407, p < 0.001) and C4b binding protein beta chain (r = 0.845, p < 0.05).

**Figure 5:**
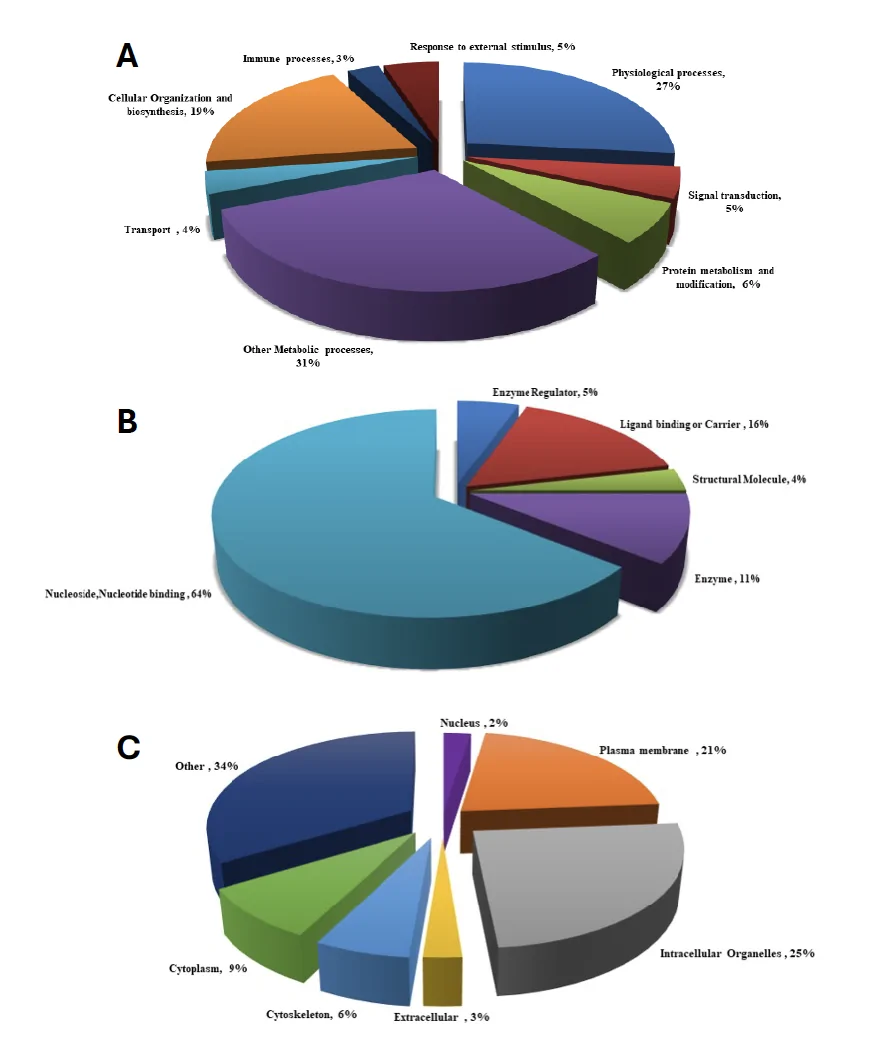
3D pie chart of the Gene Ontology of all *P. falciparum* proteins isolated from malaria samples that are involved in biological processes (A), molecular functions (B) and cellular componenents (C).

**Figure 6:**
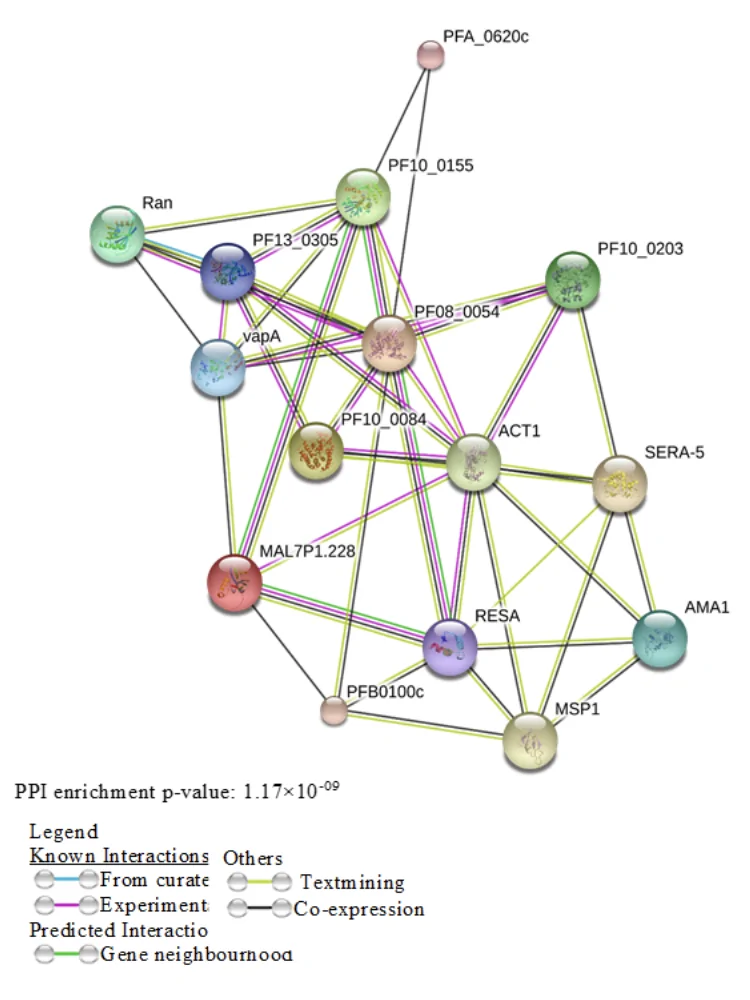
A network model delineating the P. falciparum proteins isolated from the MPs in malaria positive plasma and their associated pathways. The node colours represent MP proteins and their interactions (colour coded basis for a particular interaction).

Figure 5 presents a functional and structural overview of the proteins isolated from microparticles (MPs) of malaria-positive samples based on their biological and molecular functions or cellular components associated with the identified proteins.

while Figure 6 depicts a network model outlining the *P. falciparum* proteins detected in MPs and their related biological pathways.

## Discussion

The study identified an array of proteins from the MPs isolated from the malaria-positive samples, culture supernatant and healthy controls. The mean haemoglobin (Hb) of the studied subjects was significantly lower than that of the control group (p = 0.037). This finding agrees with a study in the Brazilian Amazon in which out of a total of 7831 people studied, individuals with malaria were seen to have the lowest Hb compared to community controls and malaria-negative febrile patients [13]. The results of our study also corroborate with the results from a study carried out in Nigeria [14]. These observations are expected because, as far back as in 1947 *Plasmodium* parasites were reported to “consume” haemoglobin during their growth within erythrocytes [15]. *P. falciparum*, for instance, is capable of digesting more than 80% of the erythrocyte haemoglobin to make space and acquire amino acids [15].

This goes to explain the significant negative correlation of Hb and parasite count (r = −0.415, p < 0.001) indicating that the greater the parasite density in a patient the lower the Hb, as was also reported in a study from Thailand [16].

Again, there was a reduction of the haematocrit (Hct) of the patients (Table 2) as compared to controls which was, however, not significant. Similar results were reported in patients with uncomplicated malaria where the authors indicated that, uncomplicated malaria was associated with milder biochemical alteration and haemolysis as opposed to complicated/severe malaria [17]. A study done at ECWA Community Health Centre, Bukuru, Jos in Nigeria, however, showed significant reduction of the haematocrit of malaria samples compared to healthy controls [14]. The mean cell volume (MCV) of the *P. falciparum* patients was significantly lower than that of the control group (Table 2). This result was unexpected because previous studies in a population near Thailand-Myanmar border indicated the opposite finding because usually, as erythrocytes are being destroyed during malaria infection, the bone marrow is stimulated to produce and release more new erythrocytes whose mean cell volume are higher than older erythrocytes [16]. Also, the mean cell haemoglobin (MCH) of patients was significantly lower than that of the control group which is because malaria parasites digest haemoglobin during their intraerythrocytic growth and infection also promotes the release of haemoglobincontaining microparticles. These processes reduce the haemoglobin content of circulating red blood cells, resulting in a lower MCH compared with healthy controls. This result, however, contradicts with the results of the population near the Thailand-Myanmar border [16].

The red blood cell distribution width (RDW) was significantly higher (p = 0.032) in the patients compared to the control group. It was, however, consistent with results from a *P. vivax* malaria study [18]. The mean RDW was 15.80 and RDW greater than 15 has been shown to be a predictive value of malaria infection, although some researchers contest this [19]. In *P. vivax* malaria however, the elevated RDW results from the initial size increase of parasitised erythrocytes which is followed by erythrocyte rapture. The same mechanism, however, cannot be said to be associated with RDW increase in *P. falciparum* infection since parasitised cells maintain their sizes [19]. An explanation for this is that malaria-infected patients often exhibit a high RDW due to the presence of a mixed population of red blood cells, including damaged, haemolysed cells and larger reticulocytes released in response to haemolysis. At the same time, chronic inflammation, iron restriction, and marrow stress produce smaller red blood cells, resulting in a reduced MCV. The combination of microcytosis and marked anisocytosis therefore explains the characteristic pattern of high RDW with low MCV in malaria infection. The elevated RDW seen in this study could result from the bone marrow’s effort to balance erythrocytes loss in malaria by releasing more new erythrocytes into circulation which also increases the macrocyte percentage [20].

Our data indicated that a significantly higher amount of all haemoglobin subunits released came from MPs of malaria positive samples although MPs from non-malaria samples also showed some amount of haemoglobin subunits. This points to an interesting phenomenon where the erythrocytes from malaria patients lose their haemoglobin to vesiculation and the vesicles that result may be eliminated by the body thereby contributing to anaemia. Table 3 shows a list of 24 RAB proteins found in the malaria samples. They include proteins which play pivotal roles in relation to MP docking, fusion and appropriate targeting of various cellular compartments [21].

Also, among the proteomics data were some erythrocyte cytoskeletal proteins which have been referred to as low abundance cytoskeletal proteins [21]. They include myosin chains, moesin, ezrin and F-actin capping proteins. Furthermore, some *P. falciparum* proteins which have been described as potential drug targets were identified in the MPs and include: enolase, Hsp 90, hypoxanthine guanine phosphoribosyl transferase, L-lactate dehydrogenase and phosphoglycerate kinase. This is because the parasite through these proteins in their pathways starts processes such as glycolysis, haemoglobin digestion and salvage of purines [22]. Tubulin Beta which is a brain antigen that can discriminate cerebral malaria from other forms of the disease was identified in MPs from 38 samples [22]. This protein chain is an antimalarial target [22].

Enolase is a glycolytic enzyme and especially essential in ATP generation in organisms that are devoid of the Krebs cycle. In malaria, surface enolase assists in parasite invasion by binding to plasminogen [23]. Enolase was found in MPs of 38 of the samples. It is known to play a crucial role in the parasite invasion of the midgut of the mosquito [23]. A study has shown that its levels are elevated in infected red blood cells compared to uninfected cells. Antibodies against merozoite surface enolase have the ability to interfere with *P. falciparum* invasion of the red cell thereby conferring a partial protection against malaria [24]. Knob-associated histidine rich protein (KAHRP) was identified in only 1 of the 43 malaria samples. KAHRP is a major component of the knob which is an electron-dense protrusion located on the membrane of infected erythrocytes. The knob is the site of adhesive interaction between infected erythrocytes and vascular endothelial cells [24].

*Plasmodium falciparum* Merozoite surface protein 1 (MSP-1) which is also called precursor to major merozoite surface antigens (PMMSA) or merozoite surface antigen 1 (MSA-1) was identified in MPs from 2 of the malaria samples [25]. This protein is the most widely studied of proteins of *P. falciparum*. MSP-1 is well conserved among *P. falciparum* isolates and hence it was identified in the MP isolated from the culture supernatant [25]. This protein binds to erythrocytes in a sialic-dependent manner suggesting it is a receptor to a ligand on erythrocyte surface permitting adhesion of *P. falciparum* [25].

Also, merozoite surface antigen 2 (MSP-2) which is a surface coat protein essential for the survival of the blood-stages of *P. falciparum* was identified [26]. This protein and its allelic form 2 were identified in MPs from one sample. Also identified in one sample was *P. falciparum* Apical membrane antigen 1(PfAMA1) which is synthesised during schizogony and transported to micronemes. PfAMA1 translocates onto the merozoite surface prior to erythrocyte invasion when it serves as an adhesion molecule playing a central role in the invasion process [26]. Ring-infected erythrocyte surface antigen (RESA), a protein expressed in early stage gametocytes, final stages of schizont and stored in dense granules within the merozoites was found in one sample. RESA binds to spectrin, its primary site on erythrocytes and is associated with the membrane of recently invaded erythrocyte but it is only evident in the cell up to 24 hrs after parasite invasion [27].

Glutamic acid-rich protein (GLURP), found in one sample, is a molecule located on the surface of merozoites and in the hepatic stage [28]. This protein is an antigen and its epitopes defined by non-repetitive sequence are thought of to be more effective antibody dependent cellular inhibition process and is believed to be involved in acquired protective immunity to malaria. These epitopes are therefore proposed to be preferred in vaccine formulations against malaria [28]. Also, *P. falciparum* actin 1(PfACT1) was expressed in the MPs of 35 samples. PfACT1 is a ubiquitously expressed protein and it is expressed throughout the *Plasmodium* lifecycle. However, its isoform PfACT 2, which is expressed only in the sexual stages of the parasite, was not isolated in any of the samples.

Heat shock 70 kDa protein (Hsp70) was expressed in 35 samples. *P. falciparum* has six Hsp70s [29]. Hsp70 proteins have molecular chaperone functions, and are involved in a number of processes including protein degradation and controlling of the activity of regulatory proteins, protein folding and protein translocation across membranes. Hsp 70 members are present in almost all cellular compartments [29] and this could account for its identification in a good number of the samples. Also, elongation factor-1 alpha was found in the MPs from 7 of the malaria positive samples. It is an abundant protein that is an essential element in eukaryotic protein translation, in *Plasmodium* species [30]. It is key in the proliferation of the blood stages of *Plasmodium* [30].

Precursors of serine-repeat antigen (SERA) proteins are synthesised in the late trophozoites [31]. Among organisms in the apicomplexan phylum and with the exception of Theileria found in cattle, *Plasmodium* species are the only organisms that the gene family translating into Serine-repeat antigen (SERA) protein has been found [31]. This protein was found in one sample. Investigation with anti-SERA antibody has established SERA protein as a target for antibody dependent cellular inhibition of *P. falciparum* development [31].

Also identified was *P. falciparum* ADP ribosylation factor 1 (PfARF1), which is activated after binding to GTP. In the secretary pathway, PfARF1 regulates vesicular biogenesis and trafficking processes [32]. It is also thought of to be involved in the transport of MSP-1 from the endoplasmic reticulum and plays a role in the activation of a calcium-signalling mechanism in the parasite [33]. Hypothetical protein identified 300 kDa antigen AG231 (Fragment) was also found in one sample. This protein was discovered in about 93% of 65 patients living in a malaria endemic area in Papua Guinea. It is found in schizonts and trophozoites. Again, *P. falciparum* S antigen proteins were found in the MPs of 2 samples. They are heat-stable antigens that are serologically diverse among varied isolates of the parasite [33].

There was significant difference in the levels of the following complement system proteins released in plasma circulating MPs between the malaria samples and that of control: complement component C6, complement C4-B, complement component C9, complement factor B and Complement C1q subcomponent subunit C. The difference between the patients and the control group in complement component C3 was, however, not significant but correlation analysis indicated a significant negative correlation between C3 released in MPs and parasite count in the malaria samples. There was significant positive correlation between C3 and haemoglobin (r = 0.457). It is known that MPs are able to bind to complement component C1q and cause the C3 to be fixed on the MPs after exposure to normal human serum through the classical pathway. These complement-fixed MPs can bind to erythrocytes and remove them from circulation [34]. It could therefore be presumed that complement-fixed MPs bound to erythrocyte might be a mechanism that explains the increase erythrocyte clearance in malaria leading to reduced haemoglobin concentration. This presumption can be related to a similar work that explains the role of MPs in complement activation relating to the pathogenesis of rheumatoid arthritis [35].

Also, Hb negatively correlated with Mannanbinding lectin serine protease 2 released in MPs (Table 10). This may be explained by the ability of this protein to activate complement and interact with phagocytes and serves as an opsonin to *Plasmodium*-infected erythrocytes facilitating the elimination of such erythrocytes from circulation and invariably causing reduction in Hb [36]. C4 binding protein beta chain in MPs from malaria samples correlated positively with parasite count. C4 binding protein is an abundant plasma protein whose natural function is to inhibit the classical and lectin pathways of complement activation. Research has shown that the fusion of oligomerisation domains of C4 binding protein to MSP-1 from *Plasmodium yoelii* improved its immunogenicity [36]. C4 binding protein beta chain released in MPs could hence be acting as an adjuvant to MSP-1 antigens thereby eliciting the appropriate immune response against the parasite. Antithrombin concentration with the MPs in the patients was significantly low compared with the controls [37]. Serum concentration of antithrombin III particularly in severe malaria is reduced [37]. This is because generally malaria is associated with the consumption of Antithrombin [38].

In terms of limitations, because convenience sampling was used in this study, further stratification of the patients and controls was not done to assess the association of parameters like age category, sex and duration of onset of symptoms on the proteomic profile of subjects. If possible, a further prospective sampling involving sex and age matched controls should be employed in future studies so that data could be stratified to address the afore-mentioned limitation.

## Conclusions

Our study demonstrates a clear up-regulation of protein content in circulating plasma microparticles (MPs) from malaria-infected individuals compared to healthy controls. Malaria-associated MPs contained a broad array of proteins, although many of the identified proteins were shared across the three malaria sample categories, suggesting conserved pathogenic processes. Notably, haemoglobin subunits were present in higher abundance within MPs from malaria patients, indicating enhanced erythrocyte damage and supporting the hypothesis that the clearance of these MPs may contribute to malaria-associated anaemia.

Proteomic analysis also revealed the presence of multiple *P. falciparum*–derived proteins within circulating MPs. Interaction enrichment showed that these parasite proteins are at least partially functionally connected, forming interaction networks rather than representing random or unrelated components. The protein–protein interaction (PPI) analysis further demonstrated that the *P. falciparum* proteins exhibited more interactions among themselves than would be expected from a random set of proteins of similar size drawn from the parasite genome. This enrichment underscores the likelihood that these proteins participate in coordinated biological processes relevant to host–parasite interactions.

Overall, these findings highlight the diagnostic and pathogenic potential of MPs during malaria infection and provide a foundation for exploring their role as biomarkers or therapeutic targets.
